# Circulation of HAdV-41 with diverse genome types and recombination in acute gastroenteritis among children in Shanghai

**DOI:** 10.1038/s41598-017-01293-3

**Published:** 2017-06-14

**Authors:** Peng Li, Lang Yang, Jiayin Guo, Wenwei Zou, Xuebin Xu, Xiaoxia Yang, Xinying Du, Shaofu Qiu, Hongbin Song

**Affiliations:** 1 Institute of Disease Control and Prevention, Academy of Military Medical Sciences, Beijing, 100071 China; 2 Shanghai Changning District Center for Disease Control and Prevention, Shanghai, 200051 China; 3 Shanghai Pudong New Area Center for Disease Control and Prevention, Shanghai, 200136 China; 4 Shanghai Center for Disease Control and Prevention, Shanghai, 200236 China

## Abstract

Human adenovirus F (HAdV-F) is one of the major causative species detected in acute gastroenteritis in children worldwide. HAdV-F is composed of serotypes 40 and 41. Most studies have reported the prevalence of HAdV-41 and focused on its epidemiologic characteristics. In this study, seventeen samples were identified as HAdV-41 out of 273 fecal specimens from children with acute diarrhea in Shanghai. Five isolates were isolated and subjected to whole genome sequencing and analysis to characterize the genetic variation and evolution. Full genome analysis revealed low genetic variation (99.07–99.92% identity) among the isolates, and InDels are observed in the E2A gene and the hexon gene compared to the reference strain NIVD103. Phylogenetic analysis showed that all isolates mainly formed two genome-type clusters but with incongruence in the trees of whole genomes and individual genes. The recombination breakpoints of the five isolates were inferred by the Recombination Detection Program (RDP) and varied in the number and location of the recombination events, indicating different evolution origins. Overall, our study highlights the genetic diversity of HAdV-41 isolates circulating in Shanghai, which may have evolved from inter-strain recombination.

## Introduction

Viral gastroenteritis is one of the leading causes of child morbidity and mortality both in developing and developed countries^1^. Approximately 1.76 million children under 5 years died worldwide^2^. A number of viruses have been reported as the principal causal pathogens of acute gastroenteritis^3, 4^. Epidemiological studies reported a higher ratio of adenoviruses in acute diarrhea in developing countries^5, 6^.

Human adenoviruses (HAdVs) are non-enveloped DNA viruses, belonging to the family Adenoviridae. More than sixty HAdV serotypes have been identified and grouped into seven species (HAdV-A to HAdV-G) based on the biological and genetic characteristics. Many adenovirus species have been associated with acute diarrhea^7–10^. Species F is specifically implicated in childhood gastroenteritis and associated with acute sporadic diarrhea and occasional outbreaks^11–14^. Adenovirus serotype 41, which belongs to species F, has been reported as the prevalent serotype in acute gastroenteritis in children and estimated to account for up to twenty percent of childhood diarrhea as the second most common etiological agent after rotavirus^12, 15, 16^. Recent reports have revealed the increasing prevalence of HAdV-41 as the enteric adenovirus serotype in acute diarrhea in China^17–19^.

Temporal and geographic changes in circulating adenoviruses are often associated with genomic variations, which could possibly cause high rates of more severe illness^20–22^. Recombination played an important role in shaping the genetic diversity of adenovirus. Studies suggest that adenovirus genomes are very dynamic and recombination contributes greatly to the evolution and emergence of new circulating adenovirus pathgoens^23–25^. However, detailed information on the genetic diversity of HAdV-41 is limited and only few complete genome sequences of HAdV-F are currently available. This study aimed to investigate genome and evolution characteristics of adenovirus serotype 41 on a genome-wide level. We have performed whole genome sequencing of five HAdV-41 isolates recovered from children with severe gastroenteritis through routine surveillance in Shanghai. Genetic variation and recombination were analyzed to provide accurate understanding of the genetic diversity and evolution relationship of these isolates, which could be important for disease control and future vaccine development.

## Results

### HAdV-41 identification and genome sequencing

Seventeen of the 273 fecal specimens were found positive for adenovirus and identified as HAdV-41 using PCR and sequencing. All positive samples were cultured in Hep2 cells. Five isolates were successfully recovered and subjected to whole-genome sequencing. The PGM sequencer produced a total of 2,94,655 reads for all samples, with an average of 58,931 reads generated for each sample. After mapping to the reference genome NIVD103, remaining reads are between 21551 and 36755. The average coverage depth for each position across the reference genome ranged from 137 to 300 (Supplementary Table S1).

### Comparative genomic analysis

**Figure 1:**
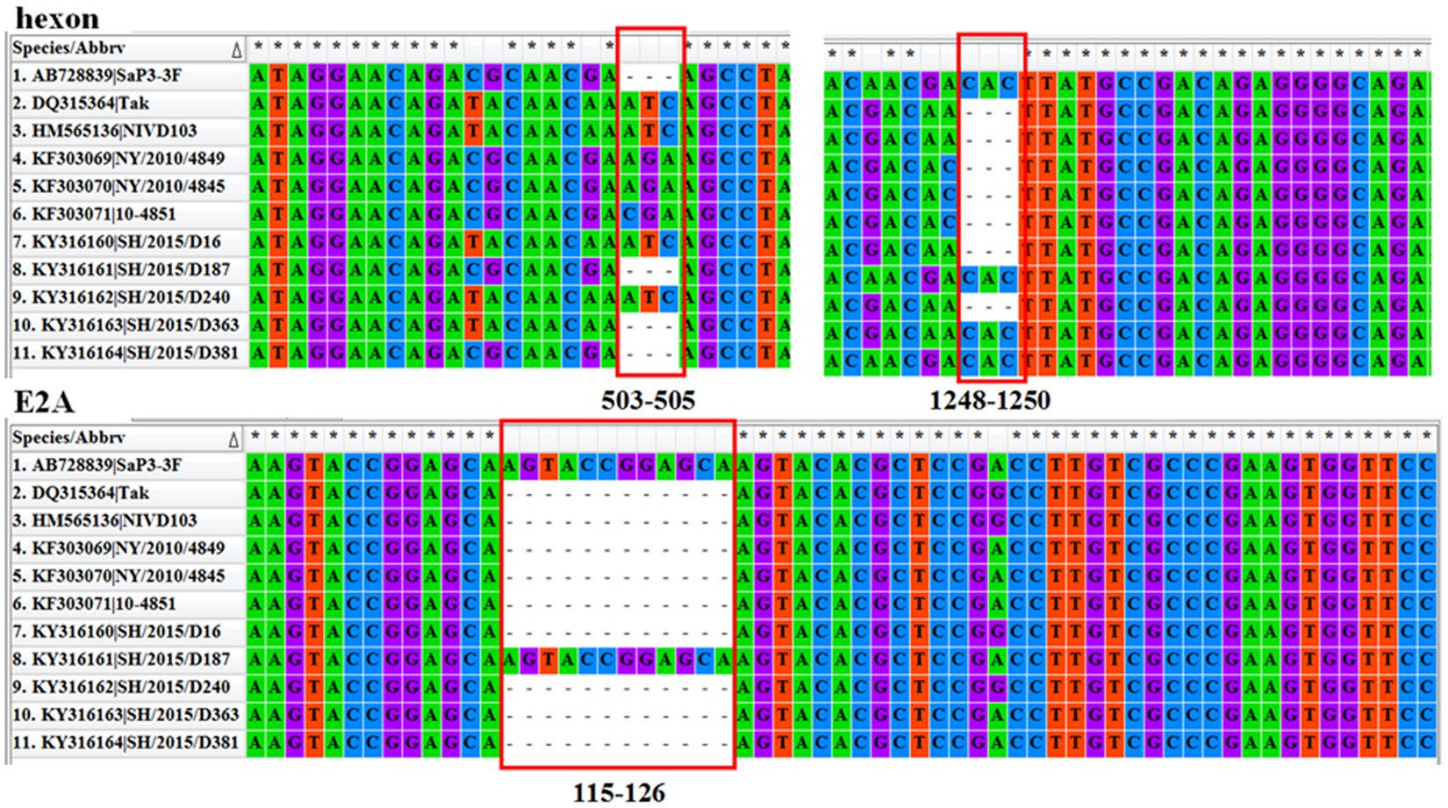
Comparative analysis of the hexon and E2A genes. Sequences of genes were aligned and visualized using MEGA6. Position of InDels compared to the reference genome NIVD103 are indicated in the figure.

Pairwise alignments revealed low-level nucleotide variations in many regions and the sequence identities varied from 98.94% to 99.74% among the five isolates. Isolates SH/2015/D16, SH/2015/D240, and SH/2015/D363 showed high similarities to the reference strain NIVD103 from China (99.75%, 99.74% and 99.45% nucleotide identity, respectively), while SH/2015/D187 and SH/2015/D381 shared significant sequence similarities to NY/2010/4845 (99.93% identity) from USA and SaP3-3F (99.88% identity) from Japan, respectively. Most of variations were single nucleotide mutations and located mainly in the coding regions (Supplementary Table S2). However, the E2A and hexon genes of the isolates presented some InDels compared to that of the reference strain NIVD103 (Fig. 1). Sequence alignment showed that E2A of SH/2015/D187 had 99.93% similarity to that of SaP3-3F and both had a 12-bp insertion (115–126, CGGAGCAAGTAC) compared to all other HAdV-41 isolates. Deduced amino acids revealed a 4 amino acids insertion of SH/2015/D187 compared to the reference genome NIVD103. SH/2015/D16 shared identical nucleotide sequence of the hexon gene with SH/2015/D240. However, SH/2015/D381, SH/2015/D187, SH/2015/D363 had a 3-bp deletion (503–505, AAT) and a 3-bp insertion (1248–1250, CAC), same as SaP3-3F, compared to SH/2015/D16, SH/2015/D240 and the reference strain NIVD103.

### Phylogenetic analysis of the complete genome and individual genes

**Figure 2:**
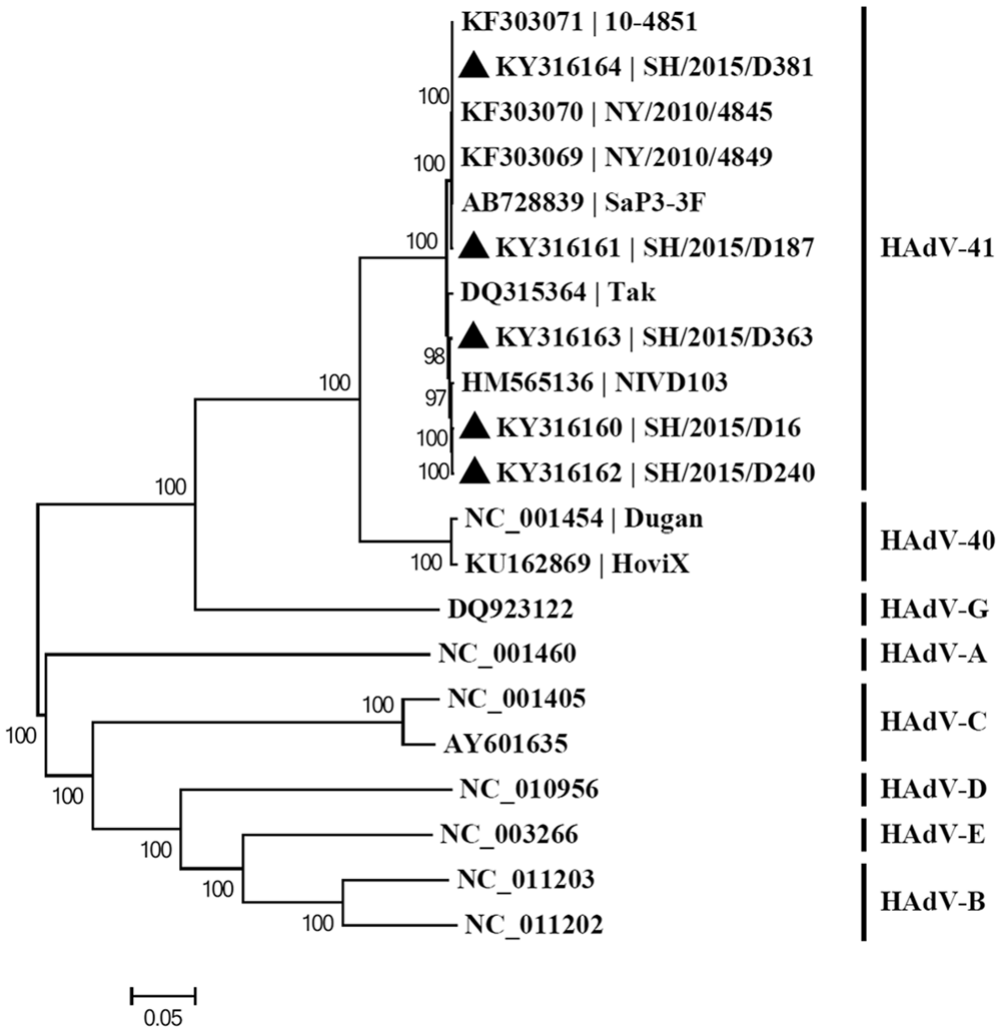
Phylogenetic tree of the five isolates, along with all available HAdV-F isolates and other representative HAdV species. The trees were constructed by neighbor-joining method using MEGA6 with 1,000 bootstrap replicates. Black triangles represent the 5 HAdV-41 isolates in this study. Numbers at tree nodes indicate percentages of bootstrap values. The GenBank accession numbers are contained in the name of isolates.

A whole-genome phylogenetic tree of the isolates in this work, together with all available HAdV-F isolates and other representative HAdV species is shown in Fig. 2. It’s noted that all five isolates (Bold Triangle) belonged to serotype 41 and showed clear divergence. SH/2015/D16, SH/2015/D240 and SH/2015/D363 formed a cluster and indicated close evolutionary relationship with the domestic strain NIVD103. However, SH/2015/D187 and SH/2015/D381 formed a distinct cluster together with isolates from USA (KF303069-KF303071) and Japan (AB728839), suggesting different evolutionary origin.

**Figure 3:**
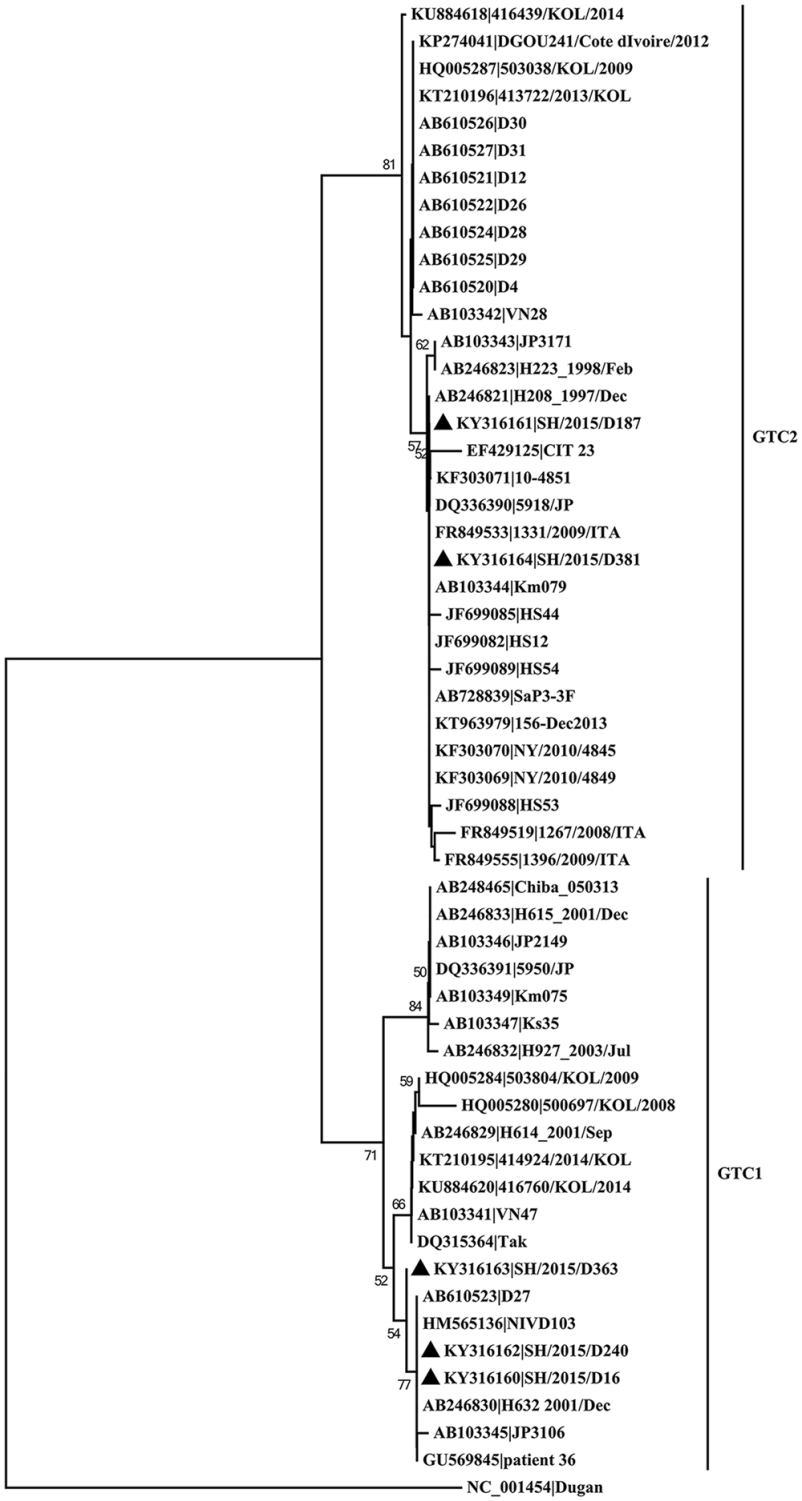
Phylogenetic tree based on the HVRs of the HAdV-41 hexon genes of reference strains from previous studies and our isolates from Shanghai. Forty-three HAdV-41 isolates with partial hexon sequences are also included and indicated with accession numbers and names.

**Figure 4:**
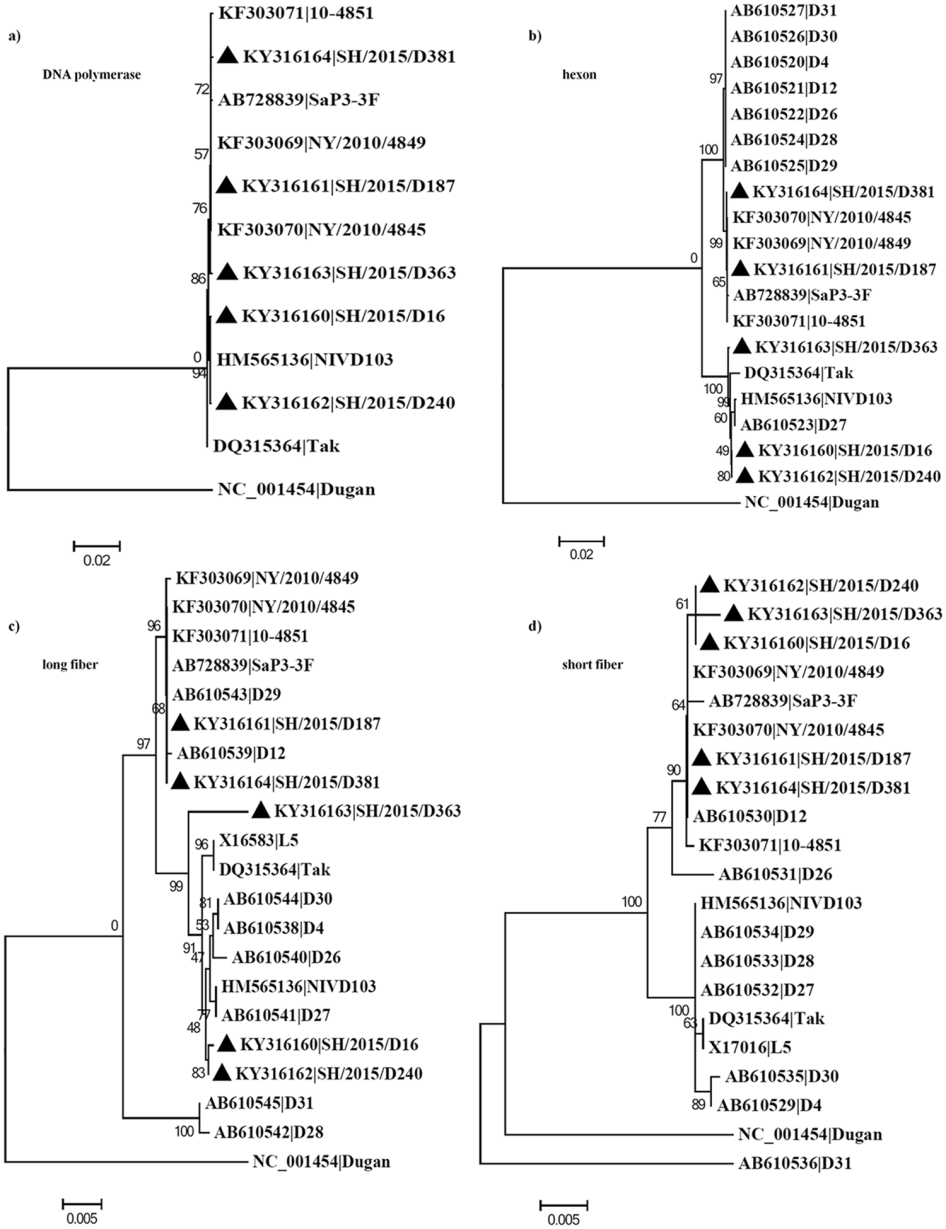
The Neighbor-Joining phylogenetic tree showing relationship of five isolates in this study to other adenoviruses inferred from (**a**) DNA polymerase, (**b**) hexon gene, (**c**) long fiber gene, and (**d**) short fiber gene.

We also constructed the neighbor-joining tree of HVRs of the hexon gene described in a previous study^15^. The result revealed that the five isolates formed two genomic type clusters (GTCs) (Fig. 3). SH/2015/D381 and SH/2015/D187 belonged to GTC2, while SH/2015/D363, SH/2015/D240 and SH/2015/D16 belonged to GTC1, indicating that there exist two distinct HAdV-41 genome types circulating in Shanghai. Phylogenetic analysis based on the entire hexon gene was in accordance with the HVRs-based tree (Fig. 4b). However, SH/2015/D363 are more divergent from other isolates belonging GTC1 in the HVR tree. It’s noteworthy that SH/2015/D363 shared same InDels in the hexon gene with SH/2015/D187 and SH/2015/D363, but fell into different clusters.

Trees of the long and short fiber genes also formed two clusters but showed incongruence with the tree of the hexon gene (Fig. 4c,d). The five isolates still fell into two clades in a subgroup of the long fiber tree but all clustered in the same clade in the short fiber tree.

Much less diversity was observed in the DNA polymerase gene (Fig. 4a), which shared >99.7% nucleotide identity among each other. Therefor these isolates did not branch out as two distinct clusters. Interestingly, SH/2015/D363 belonging two GTC1 now closely clustered with SH/2015/D187 and SH/2015/D381 from GTC2. Tree based on the penton gene was also constructed and showed similar patterns with the tree of the DNA polymerase gene (Supplementary Fig. S1). The Maximum-Likelihood and Bayesian approaches were also employed to verify phylogenetic results and both methods returned essentially the same topology as those obtained by the Neighbor-Joining method (Supplementary Figs S2 and S3).

### Recombination analysis

Recombination analysis using the RDP was performed to determine the potential evolutionary origins. Evidences for recombination varied in strength among the five isolates (Table 1). SH/2015/D16, SH/2015/D187 and SH/2015/D240 only presented one predict recombinant event. The recombination breakpoint of SH/2015/D187 was located in the E1A/E1B region. Both SH/2015/D16 and SH/2015/D240 shared a large common breakpoints located between the E2A gene and the short fiber gene. Six different recombination breakpoints were inferred in SH/2015/D363, originating from a recombination between NY/2010/4845 and NIVD103. RDP analysis results for SH/2015/D363 were consistent with the phylogenetic analysis above. However, no recombination event was detected in SH/2015/D381.

**Table 1:** Recombination events detected with RDP4 from the alignment of whole genome and individual genes used for tree construction. The average P-value of detection methods are indicated.

Recombinant	Breakpoints	Major Parent	Minor Parent	Recombination Detection Methods (Av. P-alue)						
				RDP	GENECONV	BootScan	MaxChi	Chimaera	SiScan	3Seq
**WholeGenome**										
SH/2015/D187	184–1581	SH/2015/D381	NIVD103	2.91 × 10^−09^	5.11 × 10^−07^	2.81 × 10^−09^	4.83 × 10^−03^	4.04 × 10^−02^	3.67 × 10^−05^	—
SH/2015/D16	19659–29993	NIVD103	Unkown	4.65 × 10^−05^	2.58 × 10^−05^	5.44 × 10^−04^	2.21 × 10^−10^	1.19 × 10^−08^	3.69 × 10^−31^	1.62 × 10^−05^
SH/2015/D240	19659–29993	NIVD103	Unknown	4.65 × 10^−05^	2.58 × 10^−05^	5.44 × 10^−04^	2.21 × 10^−10^	1.19 × 10^−08^	3.69 × 10^−31^	1.62 × 10^−05^
SH/2015/D363	31–1085	SH/2015/D381	NIVD103	—	1.01 × 10^−05^	2.99 × 10^−06^	4.42 × 10^−02^	—	1.64 × 10^−18^	3.11 × 10^−04^
	10012–12520	NY/2010/4845	NIVD103	4.64 × 10^−03^	1.21 × 10^−02^	5.59 × 10^−03^	1.96 × 10^−02^	1.96 × 10^−02^	3.48 × 10^−06^	1.03 × 10^−02^
	17719–19568	NY/2010/4845	NIVD103	—	3.69 × 10^−11^	2.41 × 10^−15^	4.24 × 10^−13^	1.26 × 10^−11^	5.48 × 10^−09^	—
	26452–29139	Unknown	NY/2010/4845	3.89 × 10^−03^	—	—	—	—	2.18 × 10^−05^	2.28 × 10^−03^
	31194–31825	NY/2010/4845	Unknown	8.42 × 10^−07^	2.72 × 10^−06^	5.19 × 10^−07^	7.22 × 10^−03^	—	—	1.39 × 10^−03^
	31826–33318	NY/2010/4845	NIVD103	—	1.28 × 10^−10^	—	7.53 × 10^−09^	—	—	1.37 × 10^−02^
**Long fiber**										
SH/2015/D363	1492–1538	NIVD103	Unknown	—	4.89 × 10^−06^	1.61 × 10^−06^	2.53 × 10^−02^	—	—	4.87 × 10^−04^
**Hexon**										—
SH/2015/D16	293–2067	D31	Tak	—	—	—	—	—	8.32 × 10^−17^	2.19 × 10^−02^
SH/2015/D240	293–2067	D31	Tak	—	—	—	—	—	8.32 × 10^−17^	2.19 × 10^−02^
SH/2015/D363	292–1976	D31	Tak	—	—	—	—	—	8.32 × 10^−17^	2.19 × 10^−02^

Recombination of individual genes was also analyzed. Only one Recombination event was detected within the long fiber gene of SH/2015/D363, encompassing nucleotides 1492 to 1538, which may contribute to the long branch of D363 in the long fiber gene tree (Fig. 4c). Similar recombination events spanning nearly the entire hexon gene were identified within SH/2015/D16, SH/2015/D240 and SH/2015/D363 but with weak evidence. No signals of recombination were detected in the DNA polymerase, penton and short fiber genes using RDP4 software.

**Figure 5:**
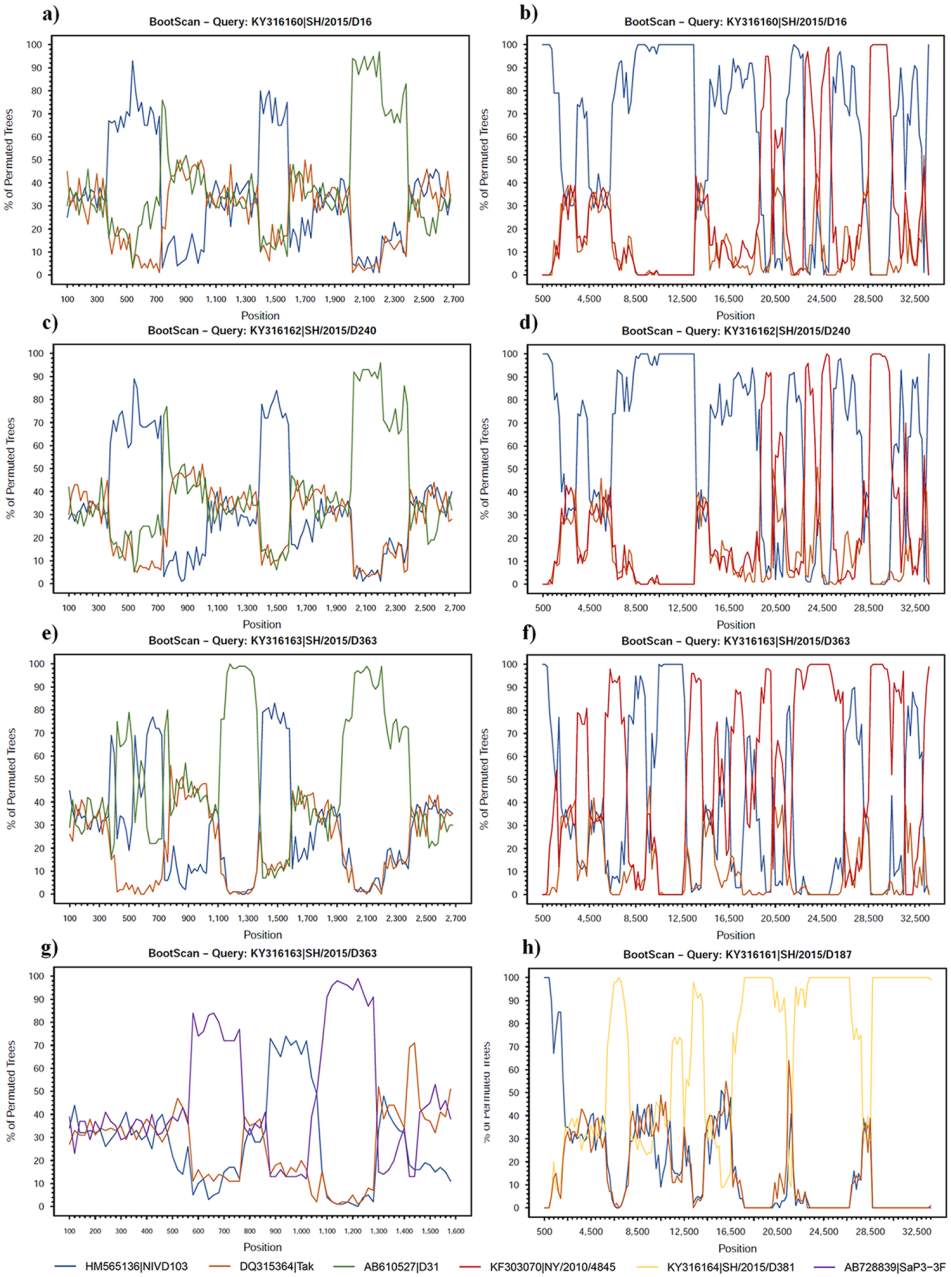
Bootscan plots of hexon, long fiber and whole genome sequences of the recombinant isolates. (**a**) Hexon of SH/2015/D16; (**b**) genome of SH/2015/D16; (**c**) hexon of SH/2015/D240; (**d**) genome of SH/2015/D240; (**e**) hexon of SH/2015/D363; (**f**) genome of SH/2015/D363; (**g**) long fiber of SH/2015/D363; and (**h**) genome of SH/2015/D187. The analysis of genes is set with a window size of 100 bp and a step size of 20 bp, while whole genomes with a window size of 1000 bp and a step size of 200 bp. The query isolates are listed above each parts of the figure, and the references are colored accordingly in the color schemes under the figure.

In addition, putative recombinants were also analyzed using SimPlot software. Previously detected recombinants were used as query, while the Major parent and Minor parent were used as references. The bootscan analysis further confirmed the recombination results of RDP analysis (Fig. 5). The similarity plot analysis clearly showed the high degree of similarity within HAdV-41 isolates (Supplementary Fig. S4), indicating recent recombination events.

## Discussion

Previous study has reported that HAdV-41 was the prevalent adenovirus serotype causing gastroenteritis in children in shanghai^18^. However, no genome information was available in this area. In the present study, we have performed whole-genome sequencing and analysis of five HAdV-41 isolates isolated from children with acute diarrhea in Shanghai. Genomic relationships were evaluated through recombination events detection using complete genome and individual gene sequences. This study showed that these isolates had different genetic variations and formed two separate clusters, indicating that two different genome types of HAdV-41 were circulating in this area.

Due to its high variable regions, the hexon gene is widely used for genome typing and relationship analysis. However, our analysis revealed the phylogenetic tree of HVRs showed small incongruence with that of the entire hexon gene in the relationship between closely related isolates, indicating the importance of variants of non HVR regions in genome typing. Phylogenetic relationship among isolates varied in different phylogenetic trees of individual genes. The two genome clusters in the trees of the hexon and short fiber gene encompassed quite different isolates. D31 and D28 belonging to GTC2 in the previous study^15^ now formed a subclade in the tree of the long fiber gene. D31 also showed huge distance in the tree of the short fiber tree from other HAdV-F isolates, but more data are needed to clarify this issue. Interestingly, most isolates belonging to GTC2 are from stool samples of patients with gastroenteritis in Asian countries, while isolates belonging to GTC1 are from stool or nasal swab samples of patients with gastroenteritis or respiratory disease and sewage samples worldwide. However, this observation needs to be investigated further with more data. And re-construction of phylogenetic trees using different molecular sequence data is important to provide full-scale resolution of the HAdV-41 relationship.

Recombination is a vital force driving evolution and contributes much to the genetic diversity of adenovirus^23, 24^. Our study showed that recombination varied between isolates circulating in Shanghai, providing new insights into HAdV-41 evolution origin. Phylogenetic analysis of the individual genes showed that the five isolates clustered into different clades, providing further evidence of recombination. It’s noteworthy that recombination events varied among these isolates with different frequency and size, which may result from selective pressure during different periods of viral evolution. Evidence of inter-strain recombination events indicated that the viral isolates probably shared a common ancestor.

The species F are particularly difficult to isolate because of slowly growing in cell culture, in contrast to other adenovirus species^26, 27^. Most of HAdV-41 positive samples in this study were not able to be inoculated and yield sufficient enough nucleotide acid for whole genome sequencing. Though HAdV-41 was reported with increasing prevalence in acute gastroenteritis, few complete genomes of HAdV-41 are currently available in the GenBank database. More complete genome sequences are needed to gain in-depth understanding of genomic epidemiology and pathogenesis of HAdV-41.

In summary, our study provided further evidence of the genetic diversity and recombination of HAdV-41 currently circulating in Shanghai. These findings highlighted the importance of whole-genome sequencing in unveiling genomic variation and recombination, which will provide crucial insights into adenovirus evolution and facilitate future vaccination strategies and development.

## Methods

### Fecal Sample Collection and HAdV-41 identification

A total of 273 fecal samples were collected from pediatric patients with acute gastroenteritis in routine surveillance during the period from January to July 2015 in Shanghai, China. Patients with ≥3 loose or watery stools within 24 h were clinically diagnosed as acute diarrhea. Nucleic acid was extracted from fecal specimens using TIANamp Virus DNA/RNA Kit (Tiangen Biotech, Beijing, China) according to the manufacture’s instruction. Extracted DNA was subjected to PCR amplification using the universal primers of the hexon gene, and followed by the specific primers of the fiber gene as previously described^28^. The PCR products were verified by sequencing and then searched against the NCBI database by BLAST. All adenovirus-41 positive samples were stored at −80 °C and then sent to our lab for further analysis. Because the fecal specimens were collected for routine surveillance during normal course of patient care and the patients were fully anonymized, written informed consent from the parents was not obtained, but the study has been approved by the Medical Ethics Committee of the Academy of Military Medical Sciences.

### Whole genome sequencing and comparative analysis

Adenovirus-41 positive samples were inoculated in Hep2 cells. Five HAdV-41 isolates were isolated and submitted for *de novo* whole genome sequencing. DNA extraction was accomplished by using QIAamp MinElute Virus Spin Kit (Qiagen) according to the manufacture’s instruction. Pure DNAs were then randomly fragmented to an average size of 350 bp by using a Covaris S200 system (Covaris Inc). Libraries were prepared by using the Ion PGM Hi-Q Sequencing Kit. Sequencing was performed on the platform Ion Torrent PGM^TM^. After filtering low quality bases and trimming the adapter, reads mapped to the reference genome NIVD103^29^ (HM565136) isolated from China were collected. *De novo* assemblies were performed using Velvet 1.2.08 and VelvetOptimiser 2.24 with k values between 21 and 151 as described previously^30^. Annotation was completed based on the annotation of NVID103 by using RATT^31^ and manually checked. Complete sequences of our isolates and all other available HAdV-F isolates were subjected to comparative genomic analysis to identify nucleotide acids changes.

### Phylogenetic analysis

Complete sequences of the hexon, fiber, DNA polymerase genes and genomes of HAdV-F and other representative HAdV species were downloaded from the NCBI database. Forty-three represented partial sequences of hexon of HAdV-41 were also obtained to analyze the genome-type cluster as described^15^. Multiple-sequence alignments of individual genes and whole genomes were performed using Clustalw2. Phylogenetic trees were constructed with the neighbor-joining (NJ) method based on the default Maximum Composite Likelihood approach using MEGA6^32^. A bootstrap resampling process of 1000 replications was used to assess robustness of individual nodes of the phylogenies. Dugan (NC_001454) of HAdV-40 serotype was used as an outgroup. Additional maximum-likelihood (ML) and Bayesian trees were also reconstructed verify phylogenetic relationship using MEGA6 and MrBayes, respectively. The ML trees were inferred based on the General Time Reversible model with 1000 bootstrap values, while the Bayesian trees inferred using GTR substitution model with gamma-distributed rate variation for 80,000 iterations. The phylogenetic relationships obtained from the NJ tree were in accordance with those from trees reconstructed by the maximum-likelihood and Bayesian inference.

### Recombination analysis

Sequence alignments of genomes and genes used in the phylogenetic analysis were utilized to assess recombination events among different HAdV-41 isolates using the program suite in RDP4^33^. Multiple methods in its default mode, such as RDP, GENECONV, BootScan, MaxChi, Chimaera, SiScan, 3Seq, LARD, PhylPro, were utilized to predict potential recombination events between the input sequences. Only those supported by at least two of nine methods are considered as significant recombination events. SimPlot 3.5.1^34^ was employed to further investigate and visualize those potential recombination events with default settings.

### Nucleotide sequence accession numbers

Genome sequences of the five isolates were submitted to GenBank/NCBI database under the following accession number: KY316160 (SH/2015/D16), KY316161 (SH/2015/D187), KY316162 (SH/2015/D240), KY316163 (SH/2015/D363) and KY316164 (SH/2015/D381). The following complete adenovirus genome available in GenBank were used: HAdV-41 (KF303069 - KF303071, AB728839, DQ315364, HM565136), HAdV-40 (NC_001454, KU162869), HAdV-A (NC_001460), HAdV-B (NC_011202, NC_011203), HAdV-C (NC_001405, AY601635), HAdV-D (NC_010956), HAdV-E (NC_003266), HAdV-G (DQ923122).

## Electronic supplementary material

Supplementary materials
