# Supplementary material for: Circulation of HAdV-41 with diverse genome types and recombination in acute gastroenteritis among children in Shanghai
Source: Sci Rep. 2017 Jun 14;7:3548. doi: 10.1038/s41598-017-01293-3 (PMC5471248; doi:10.1038/s41598-017-01293-3)
Supplement: Supplementary file 1 — Supplementary materials [file 41598_2017_1293_MOESM1_ESM.pdf]

Circulation of Human Adenovirus-41 with diverse genome types and recombination in acute gastroenteritis among children in Shanghai

Peng Li[1#], Lang Yang[1#], Jiayin Guo[2#], Wenwei Zou[3#], Xuebin Xu[4#], Xiaoxia Yang[1], Xinying Du[1], Shaofu Qiu[1*] AND Hongbin Song[1*]

[1]Institute of Disease Control and Prevention, Academy of Military Medical Sciences, Beijing, China

[2]Shanghai Changning District Center for Disease Control and Prevention, Shanghai, 200051

[3]Shanghai Pudong New Area Center for Disease Control and Prevention, Shanghai, 200136

[4]Shanghai Center for Disease Control and Prevention, Shanghai, China

[#]These authors contributed equally to this work.

[*]Address correspondence to Shaofu Qiu, qiushf0613@hotmail.com or Hongbin Song, hongbinsong@263.net

Institute of Disease Control and Prevention, Academy of Military Medical Sciences, 20 DongDa Street, Fengtai District, Beijing 100071, China.

Tel: (86)-10-66948475

Fax: (86)-10-66948475

Supplementary Figure S1. The neighbor-Joining tree based on the penton gene

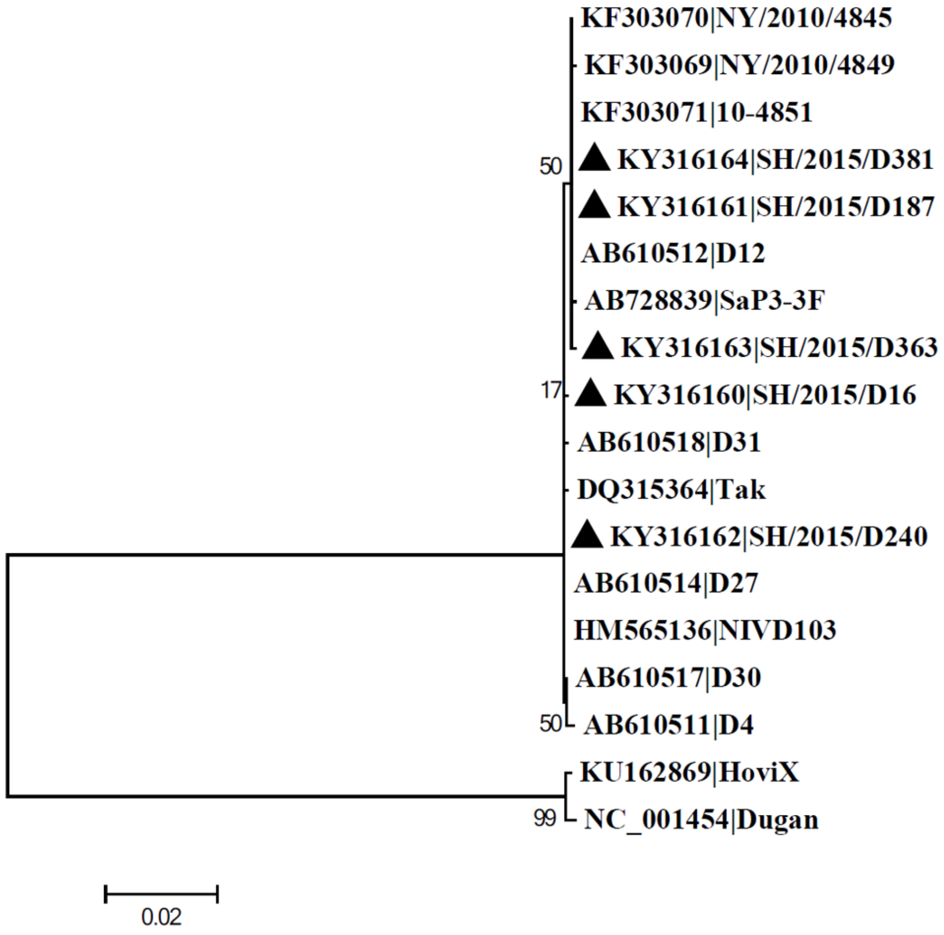

Supplementary Figure S2. The Maximum-Likelihood tree based on the whole genome (a), hexon (b), DNA po1lymerase (c), long fiber (d) and short fiber (e). The ML trees were inferred based on the General Time Reversible model with 1000 bootstrap values using MEGA6.

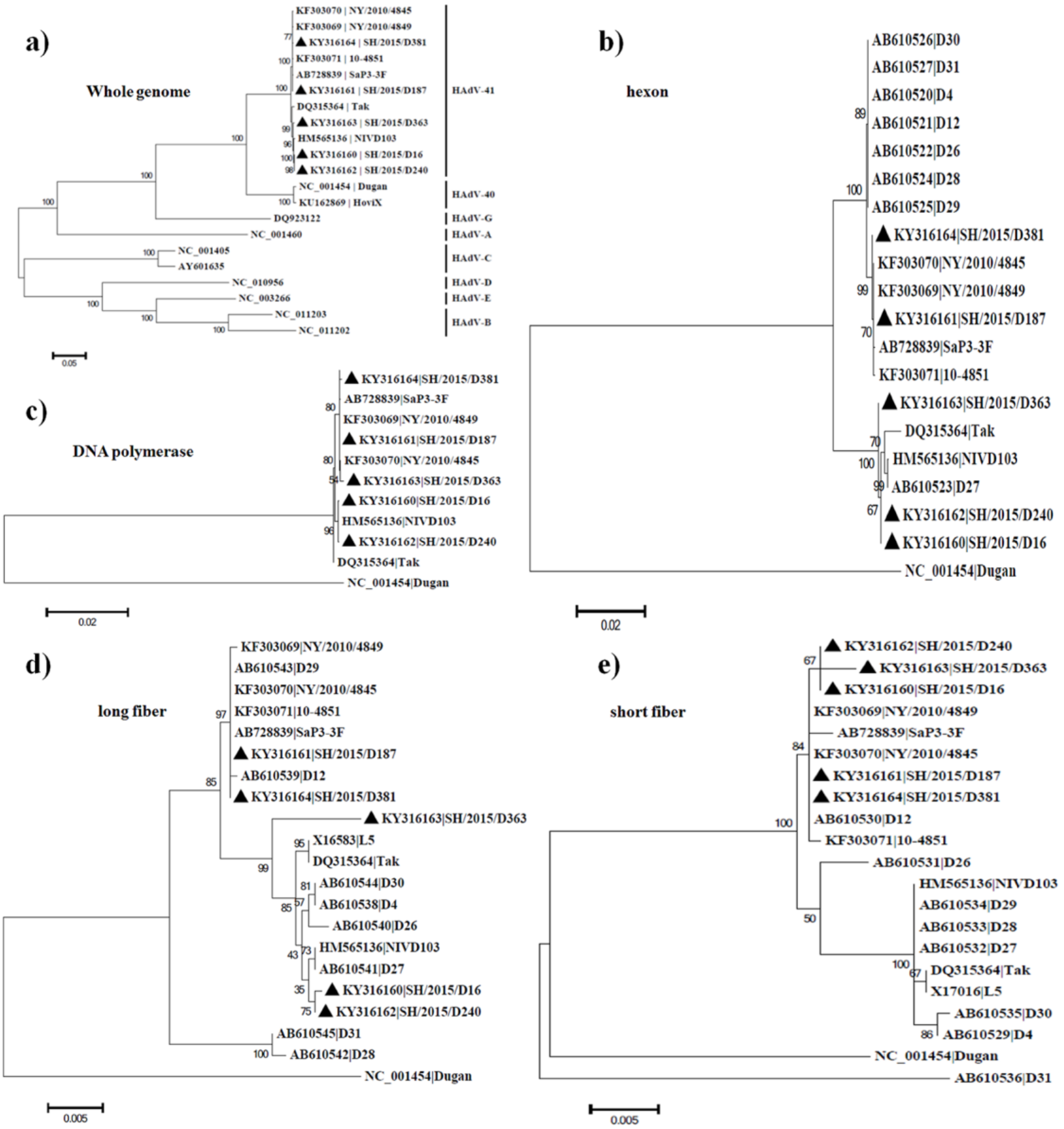

Supplementary Figure S3. The Bayesian-inferred tree based on the whole genome (a), hexon (b), DNA polymerase (c), long fiber (d) and short fiber (e). The Bayesian trees were inferred using GTR substitution model with gamma-distributed rate variation for 80,000 iterations using MrBayes.

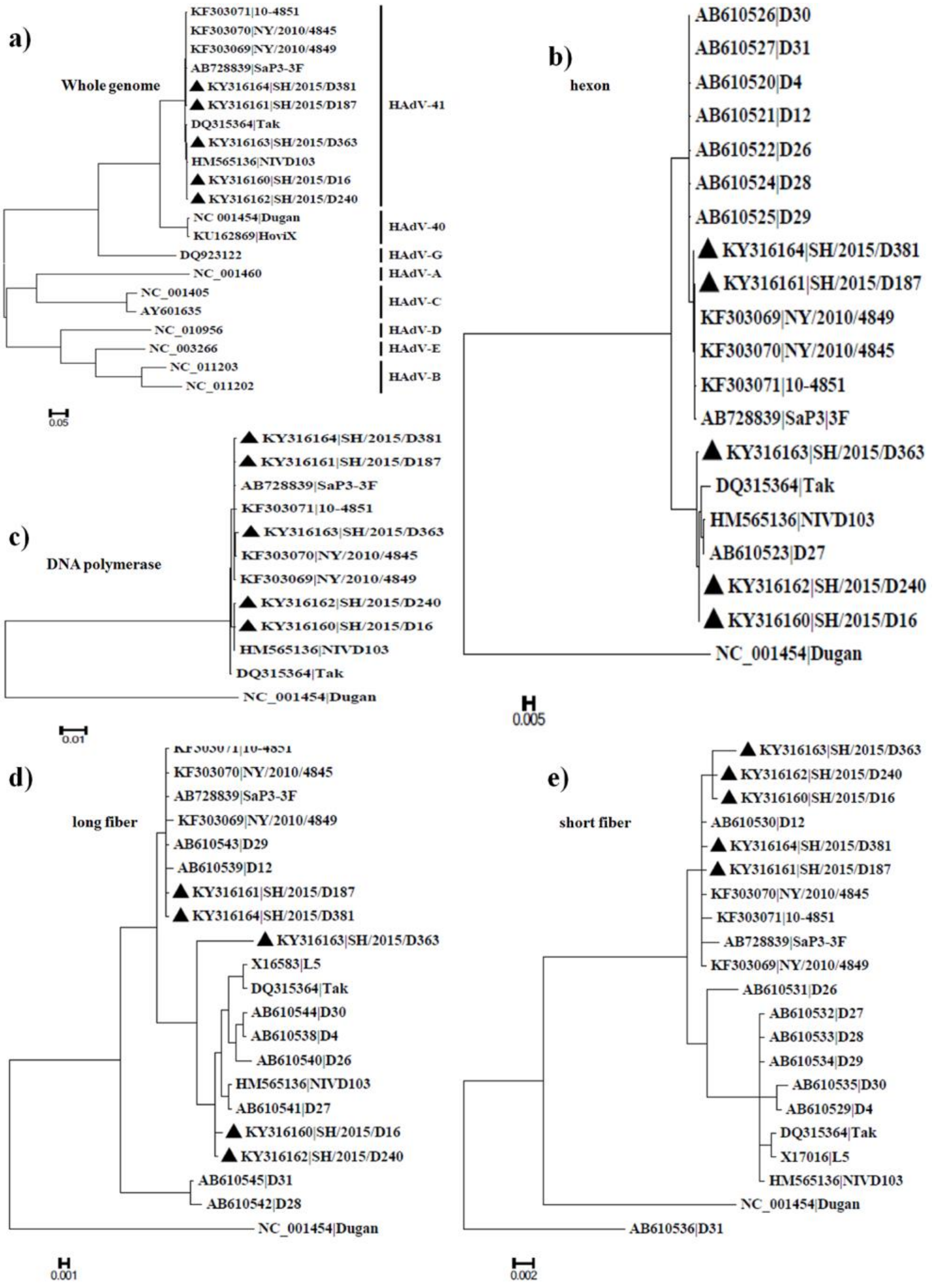

Supplementary Figure S4. Similarity plots of hexon, long fiber and whole genome sequences of the recombinant isolates. (a) hexon of SH/2015/D16; (b) genome of SH/2015/D16; (c) hexon of SH/2015/D240; (d) genome of SH/2015/D240; (e) hexon of SH/2015/D363; (f) genome of SH/2015/D363; (g) long fiber of SH/2015/D363; and (h) genome of SH/2015/D187.

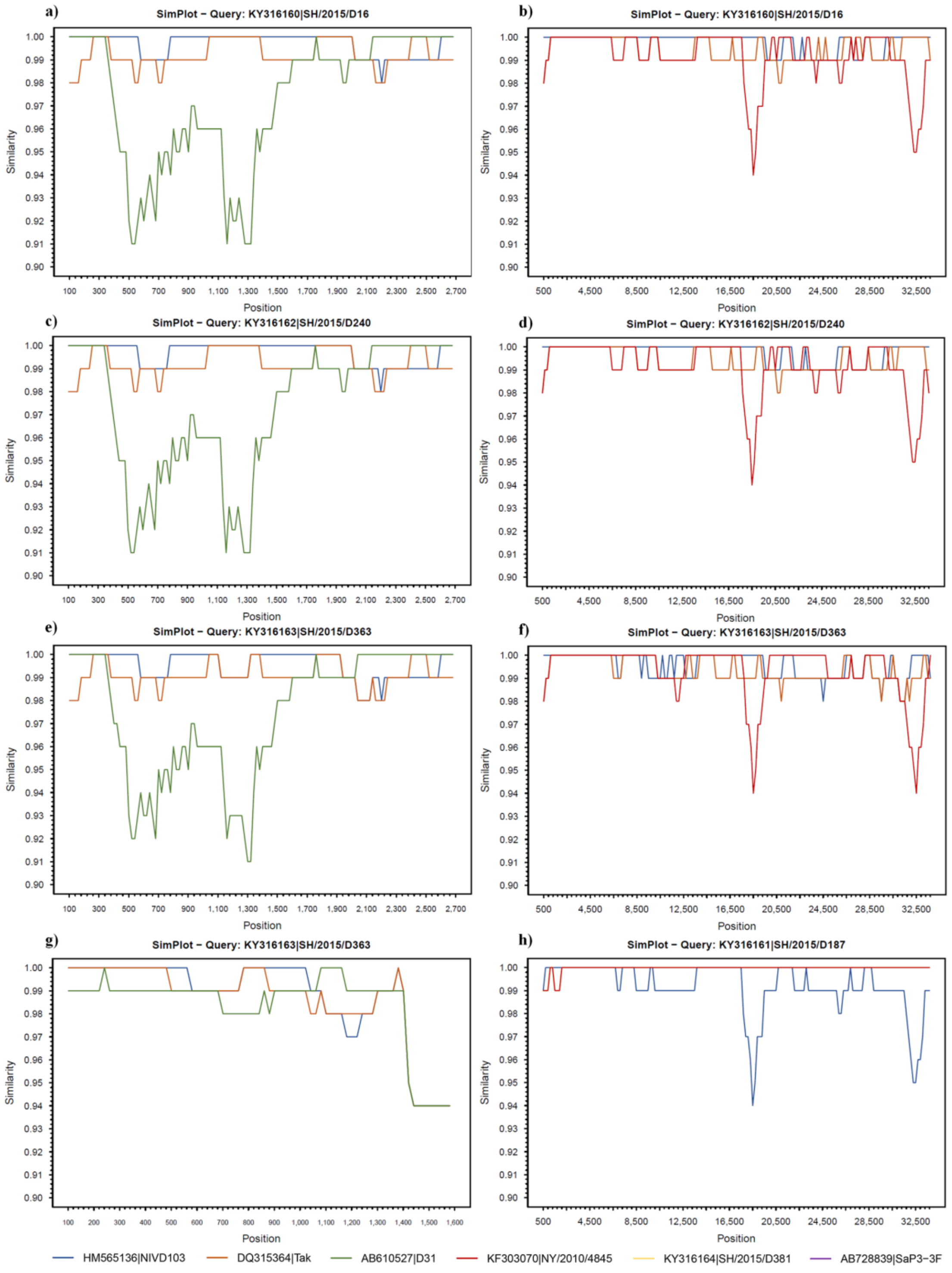

Supplementary Table S1. General features of sequenced reads of the samples

Sample	Acc. No.	No. of raw reads	No. of viral reads	Expected coverage
SH/2015/D16	KY36160	61471	31865	178
SH/2015/D18	KY36161	42861	21551	137
SH/2015/D240	KY36162	59118	22604	143
SH/2015/D363	KY36163	63040	33114	210
SH/2015/D381	KY36164	68165	36755	300

Supplementary Table S2 comparative genomic analysis of the five strains with the reference genome NIVD103

Location	Region	Gene	Nucleotide Change					
			NIVD103	SH/2015/D16	SH/2015/D	SH/2015/D240	SH/2015/D3	SH/2015/D3
62	Non-coding	-	C	T	C	T	T	C
69	Non-coding	-	T	A	T	A	A	T
77	Non-coding	-	A	T	A	T	T	A
86	Non-coding	-	T	G	T	G	G	T
115	Non-coding	-	T	C	T	C	C	T
125	Non-coding	-	T	C	T	C	C	T
172	Non-coding	-	A	A	A	A	A	-
187	Non-coding	-	G	G	G	G	G	-
243	Non-coding	-	C	T	T	T	T	C
284	Non-coding	-	G	A	A	A	A	G
324	Non-coding	-	G	A	G	A	A	G
337	Non-coding	-	G	A	G	A	A	G
361	Non-coding	-	G	G	G	G	G	A
453	Non-coding	-	C	G	G	G	G	C
481	Non-coding	-	C	T	T	T	T	C
502	CDS	E1A	G	A	A	A	A	G
517	CDS	E1A	G	T	T	T	T	G
561	CDS	E1A	C	C	C	C	C	T
571	CDS	E1A	T	C	C	C	C	T
598	CDS	E1A	G	A	A	A	A	G
635	CDS	E1A	G	C	C	C	C	G
638	CDS	E1A	T	T	C	T	T	T
818	CDS	E1A	G	A	G	A	A	G
1079	Non-coding	-	G	-	G	-	-	G
1352	Non-coding	-	C	C	C	C	G	G
1554	CDS	E1B 19K	T	C	C	C	C	T
1608	CDS	E1B 19K	T	T	A	T	T	T
1761	CDS	E1B 19K	A	G	G	G	G	G
1788	CDS	E1B 19K	C	C	T	C	C	C
1894	CDS	E1B 19K	G	G	A	G	G	G
1895	CDS	E1B 19K	A	A	G	A	A	A
1900	CDS	E1B 19K	-	-	-	G	-	-
1900	CDS	E1B 19K	-	-	-	A	-	-

1900	CDS	E1B 19K	-	-	-	G	-	-
2058	CDS	E1B 55K	C	C	C	G	C	C
3487	CDS	IX	C	T	C	C	C	C
3816	CDS	IVa2	C	T	C	T	C	C
3843	CDS	IVa2	G	G	G	G	A	A
4620	CDS	IVa2	G	G	T	G	G	G
5128	CDS	E2B DNA pol	G	G	G	G	G	T
6314	CDS	E2B DNA pol	G	G	A	G	A	A
6441	CDS	E2B DNA pol	C	T	T	T	T	T
6658	CDS	E2B DNA pol	C	C	C	C	T	C
6740	CDS	E2B DNA pol	A	A	G	A	G	G
6872	CDS	E2B DNA pol	G	G	G	G	G	A
6914	CDS	E2B DNA pol	C	T	C	T	T	C
6959	CDS	E2B DNA pol	C	C	T	C	T	T
7316	CDS	E2B DNA pol	C	T	C	T	C	C
7319	CDS	E2B DNA pol	C	C	T	C	T	T
7369	CDS	E2B DNA pol	C	C	C	C	T	C
7388	CDS	E2B DNA pol	G	G	G	A	G	G
7417	CDS	E2B DNA pol	T	G	T	T	T	T

8195	CDS	E2B DNA pol	A	G	A	G	G	A
8797	CDS	E2B pretermi nal protein	G	A	G	A	G	G
8904	CDS	E2B pretermi nal protein	G	A	G	A	A	G
8938	CDS	E2B pretermi nal protein	C	T	C	T	T	C
9016	CDS	E2B pretermi nal protein	G	A	G	A	G	G
9125	CDS	E2B pretermi nal protein	T	G	T	G	G	T
9325	CDS	E2B pretermi nal protein	A	G	G	G	G	G
9367	CDS	E2B pretermi nal protein	A	T	A	T	A	A
9739	CDS	E2B pretermi nal protein	G	A	G	A	G	G
9898	CDS	E2B pretermi nal protein	G	A	G	A	G	G
10038	Non-coding	-	T	T	T	T	A	T
10039	Non-coding	-	G	G	G	G	T	G
10041	Non-coding	-	A	A	A	A	G	A
10375	CDS	52-55K	T	C	T	C	C	T
10512	CDS	52-55K	C	T	C	T	C	C
10545	CDS	52-55K	A	A	C	A	A	A
10639	CDS	52-55K	C	C	C	C	T	C
10703	CDS	52-55K	A	A	A	A	G	A

10821	CDS	52-55K	G	A	G	A	A	G
10827	CDS	52-55K	A	G	A	G	G	A
10908	CDS	52-55K	G	A	G	A	A	G
10966	CDS	52-55K	T	C	T	C	C	T
10972	CDS	52-55K	C	T	C	T	T	C
11088	CDS	52-55K	T	C	T	C	C	T
11242	CDS	52-55K	G	A	G	A	G	G
11301	CDS	52-55K	G	A	G	A	G	G
11340	CDS	52-55K	C	T	C	T	C	C
11365	CDS	52-55K	G	A	G	-	G	G
11366	CDS	52-55K	A	G	A	-	A	A
11367	CDS	52-55K	G	G	G	-	G	G
11690	CDS	IIIa	A	G	A	G	G	A
11744	CDS	IIIa	C	T	C	T	T	C
11750	CDS	IIIa	C	T	C	T	T	C
11804	CDS	IIIa	G	G	G	G	A	G
11906	CDS	IIIa	A	G	A	G	G	A
11930	CDS	IIIa	C	T	C	T	T	C
11984	CDS	IIIa	C	C	A	C	C	A
11996	CDS	IIIa	C	T	C	T	T	C
12020	CDS	IIIa	A	G	A	G	G	A
12144	CDS	IIIa	C	T	C	T	T	C
12155	CDS	IIIa	C	T	C	T	T	C
12185	CDS	IIIa	G	C	G	C	C	G
12239	CDS	IIIa	A	C	A	C	C	A
12308	CDS	IIIa	G	T	G	T	T	G
12338	CDS	IIIa	C	T	C	T	T	C
12697	CDS	IIIa	T	C	T	C	T	T
12703	CDS	IIIa	C	T	C	T	C	C
12805	CDS	IIIa	G	C	G	C	G	G
13071	CDS	IIIa	C	C	T	C	T	T
13118	CDS	IIIa	A	G	A	G	A	A
13156	Non-coding	-	A	T	A	T	A	A
13159	Non-coding	-	T	A	A	A	A	A
13163	Non-coding	-	A	-	A	-	A	A
13164	Non-coding	-	A	-	A	-	A	A
13165	Non-coding	-	A	-	A	-	A	A
13166	Non-coding	-	A	-	A	-	A	A
13167	Non-coding	-	A	-	A	-	A	A
13168	Non-coding	-	A	-	A	-	A	A
13169	Non-coding	-	A	-	A	-	A	A
13170	Non-coding	-	A	-	A	-	A	A
13171	Non-coding	-	A	-	A	-	A	A
13172	Non-coding	-	A	-	-	-	A	A
13220	Non-coding	-	-	C	C	C	-	C

13221	Non-coding	-	T	C	C	C	C	C
13693	CDS	penton base	C	C	T	C	T	T
13699	CDS	penton base	A	A	G	A	G	G
13952	CDS	penton base	C	C	C	C	T	C
14003	CDS	penton base	T	C	C	C	C	C
14755	CDS	penton base	C	T	C	C	C	C
14798	CDS	pVII	C	T	T	T	T	T
14926	CDS	pVII	G	C	C	C	C	C
15157	CDS	pVII	G	A	G	G	G	G
15245	CDS	pVII	G	A	G	A	G	G
15269	CDS	pVII	A	G	G	G	G	G
15526	CDS	V	A	G	G	G	G	G
15709	CDS	V	C	C	C	C	T	C
15734	CDS	V	T	C	C	C	C	C
15896	CDS	V	T	C	C	C	C	C
16034	CDS	V	T	C	C	C	C	C
16100	CDS	V	T	G	G	G	G	G
16101	CDS	V	G	C	C	C	C	C
16140	CDS	V	C	A	A	A	A	A
16177	CDS	V	G	T	T	T	T	T
16190	CDS	V	A	G	A	G	G	A
16337	CDS	V	T	C	C	C	C	C
16385	CDS	V	T	C	C	C	C	C
17035	CDS	VI	T	T	C	T	C	C
17161	CDS	VI	C	T	C	T	C	C
17176	CDS	VI	T	C	C	C	C	C
17260	CDS	VI	C	C	C	C	C	T
17305	CDS	VI	G	A	G	A	G	G
17347	CDS	VI	A	G	G	G	G	G
17348	CDS	VI	A	G	G	G	G	G
17398	CDS	VI	C	C	T	C	T	T
17528	Non-coding	-	A	G	G	G	G	G
17672	CDS	hexon	C	T	T	T	T	T
17687	CDS	hexon	A	G	G	G	G	G
17738	CDS	hexon	T	C	C	C	C	C
18032	CDS	hexon	G	G	A	G	G	A
18038	CDS	hexon	T	T	A	T	T	A
18042	CDS	hexon	T	T	C	T	T	C
18060	CDS	hexon	A	G	G	G	G	G
18062	CDS	hexon	T	T	C	T	T	C
18071	CDS	hexon	T	G	T	G	G	T

18092	CDS	hexon	T	T	C	T	T	C
18093	CDS	hexon	A	A	G	A	A	G
18098	CDS	hexon	A	A	G	A	A	G
18101	CDS	hexon	T	T	-	T	-	-
18102	CDS	hexon	C	C	-	C	-	-
18103	CDS	hexon	A	A	-	A	-	-
18125	CDS	hexon	C	C	A	C	C	A
18149	CDS	hexon	T	T	C	T	T	C
18176	CDS	hexon	C	C	T	C	C	T
18180	CDS	hexon	G	G	A	G	G	A
18183	CDS	hexon	C	C	G	C	C	G
18185	CDS	hexon	A	A	C	A	A	C
18203	CDS	hexon	G	G	A	G	G	A
18204	CDS	hexon	C	C	T	C	C	T
18230	CDS	hexon	A	G	A	G	G	A
18269	CDS	hexon	C	T	T	T	T	T
18278	CDS	hexon	T	T	C	T	T	C
18280	CDS	hexon	G	G	A	G	G	A
18284	CDS	hexon	C	C	T	C	C	T
18290	CDS	hexon	G	G	A	G	G	A
18345	CDS	hexon	C	C	G	C	C	G
18349	CDS	hexon	G	G	A	G	G	A
18361	CDS	hexon	A	A	T	A	A	T
18386	CDS	hexon	A	G	G	G	G	G
18389	CDS	hexon	C	C	T	C	C	T
18393	CDS	hexon	T	T	G	T	T	G
18395	CDS	hexon	C	C	T	C	C	T
18450	CDS	hexon	A	A	G	A	A	G
18476	CDS	hexon	G	G	A	G	G	A
18497	CDS	hexon	G	G	A	G	G	A
18524	CDS	hexon	T	C	C	C	C	C
18527	CDS	hexon	C	C	T	C	C	T
18563	CDS	hexon	T	T	C	T	T	C
18578	CDS	hexon	A	A	G	A	A	G
18632	CDS	hexon	A	A	G	A	A	G
18650	CDS	hexon	C	C	T	C	C	T
18653	CDS	hexon	A	A	T	A	A	T
18680	CDS	hexon	T	T	C	T	T	C
18722	CDS	hexon	G	G	A	G	G	A
18753	CDS	hexon	C	C	T	C	C	T
18803	CDS	hexon	C	C	T	C	T	T
18815	CDS	hexon	G	G	A	G	G	G
18818	CDS	hexon	C	C	A	C	C	A
18821	CDS	hexon	T	T	C	T	T	C
18822	CDS	hexon	G	G	A	G	G	A

18825	CDS	hexon	C	C	G	C	C	G
18826	CDS	hexon	A	A	G	A	A	G
18829	CDS	hexon	C	C	A	C	C	A
18839	CDS	hexon	A	A	G	A	A	G
18843	CDS	hexon	G	G	A	G	G	A
18846	CDS	hexon	A	A	G	A	A	G
18848	CDS	hexon	-	-	C	-	C	C
18848	CDS	hexon	-	-	A	-	A	A
18848	CDS	hexon	-	-	C	-	C	C
18875	CDS	hexon	A	A	G	A	A	G
18884	CDS	hexon	C	C	T	C	C	T
18938	CDS	hexon	C	C	T	C	C	T
18950	CDS	hexon	T	T	C	T	T	C
18951	CDS	hexon	T	T	C	T	T	C
18956	CDS	hexon	C	C	T	C	C	T
18974	CDS	hexon	G	G	A	G	G	A
18998	CDS	hexon	G	G	A	G	G	A
19058	CDS	hexon	C	C	T	C	C	T
19076	CDS	hexon	C	T	C	T	T	C
19154	CDS	hexon	A	A	G	A	A	G
19238	CDS	hexon	C	G	G	G	G	G
19244	CDS	hexon	G	G	T	G	G	T
19457	CDS	hexon	C	C	T	C	C	T
19508	CDS	hexon	A	A	C	A	A	C
19613	CDS	hexon	C	C	T	C	T	T
19691	CDS	hexon	T	C	C	C	C	C
19706	CDS	hexon	T	C	C	C	C	C
19835	CDS	hexon	T	C	C	C	C	C
19874	CDS	hexon	C	T	C	T	T	C
20072	CDS	hexon	T	T	C	T	T	C
20198	CDS	hexon	C	T	T	T	T	T
20412	CDS	protease	T	C	C	C	C	C
20421	CDS	protease	C	T	T	T	T	T
20434	CDS	protease	C	T	T	T	T	T
20441	CDS	protease	T	C	C	C	C	C
20467	CDS	protease	G	A	A	A	A	A
20572	CDS	protease	C	G	C	G	G	C
20614	CDS	protease	A	G	G	G	G	G
20680	CDS	protease	C	C	T	C	C	T
20716	CDS	protease	C	T	T	T	T	T
20734	CDS	protease	G	A	A	A	A	A
20848	CDS	protease	G	A	T	A	A	T
20875	CDS	protease	T	C	C	C	C	C
20878	CDS	protease	A	G	A	G	A	A
20918	CDS	protease	C	T	T	T	T	T

21059	Non-coding	-	-	T	T	T	-	-
21059	Non-coding	-	-	T	-	T	-	-
21059	Non-coding	-	-	T	-	T	-	-
21059	Non-coding	-	-	T	-	T	-	-
21075	CDS	E2A DBP	C	T	T	T	T	T
21223	CDS	E2A DBP	A	G	G	G	G	G
21286	CDS	E2A DBP	G	G	G	G	A	G
21359	CDS	E2A DBP	C	T	T	T	T	T
21439	CDS	E2A DBP	T	C	C	C	C	C
21471	CDS	E2A DBP	C	T	T	T	T	T
21718	CDS	E2A DBP	G	A	G	A	A	G
21880	CDS	E2A DBP	G	A	G	A	A	G
21958	CDS	E2A DBP	C	T	T	T	T	T
22051	CDS	E2A DBP	T	C	C	C	C	C
22166	CDS	E2A DBP	C	T	C	T	C	C
22195	CDS	E2A DBP	T	C	T	C	T	T
22350	CDS	E2A DBP	C	C	T	C	T	T
22378	CDS	E2A DBP	-	-	C	-	-	-
22378	CDS	E2A DBP	-	-	T	-	-	-
22378	CDS	E2A DBP	-	-	C	-	-	-
22378	CDS	E2A DBP	-	-	C	-	-	-
22378	CDS	E2A DBP	-	-	G	-	-	-
22378	CDS	E2A DBP	-	-	G	-	-	-
22378	CDS	E2A DBP	-	-	T	-	-	-
22378	CDS	E2A DBP	-	-	A	-	-	-

22378	CDS	E2A DBP	-	-	C	-	-	-
22378	CDS	E2A DBP	-	-	T	-	-	-
22378	CDS	E2A DBP	-	-	T	-	-	-
22378	CDS	E2A DBP	-	-	G	-	-	-
22396	CDS	E2A DBP	C	C	T	C	T	T
22413	CDS	E2A DBP	G	G	A	G	A	A
22454	CDS	E2A DBP	A	C	A	A	A	A
22455	CDS	E2A DBP	C	G	C	C	C	C
22584	CDS	100K	T	C	C	C	C	C
22830	CDS	100K	T	C	C	C	C	C
22941	CDS	100K	C	T	T	T	T	T
23103	CDS	100K	A	G	A	G	A	A
23116	CDS	100K	C	C	C	C	A	C
23117	CDS	100K	A	A	A	A	C	A
23253	CDS	100K	T	A	T	T	T	T
23328	CDS	100K	T	C	C	C	C	C
23358	CDS	100K	T	C	C	C	C	C
23375	CDS	100K	G	C	C	C	C	C
23428	CDS	100K	T	C	C	C	C	C
23431	CDS	100K	G	A	A	A	A	A
23553	CDS	100K	C	C	T	C	C	T
23625	CDS	100K	T	C	C	C	C	C
23643	CDS	100K	T	T	T	C	T	T
23772	CDS	100K	C	C	T	C	C	T
23865	CDS	100K	A	A	G	A	G	G
23883	CDS	100K	A	A	A	A	G	A
23916	CDS	100K	C	C	T	C	T	T
23982	CDS	100K	C	T	C	C	C	C
23991	CDS	100K	T	C	T	C	T	T
23997	CDS	100K	C	T	C	T	C	C
24033	CDS	100K	C	C	T	C	T	T
24148	CDS	100K	T	T	C	T	C	C
24173	CDS	100K	G	G	A	G	A	A
24186	CDS	100K	G	G	A	G	A	A
24218	CDS	100K	G	G	A	G	A	A
24294	CDS	100K	G	G	G	A	G	G
24360	CDS	100K	A	A	G	A	G	G
24363	CDS	100K	C	C	T	C	T	T

24745	CDS	100K	C	T	T	T	T	T
24751	CDS	100K	A	G	G	G	G	G
24812	CDS	100K	C	T	C	T	T	C
24898	Non-coding	-	C	C	-	-	G	-
24899	Non-coding	-	A	A	-	-	C	-
24900	Non-coding	-	C	C	-	-	C	-
24911	Non-coding	-	-	-	-	-	G	-
24947	Non-coding	-	-	-	-	-	T	-
24949	Non-coding	-	T	C	C	C	C	C
25133	Non-coding	-	T	T	C	T	T	C
25218	Non-coding	-	C	T	T	T	T	T
25289	Non-coding	-	C	T	C	T	T	C
25339	Non-coding	-	G	G	A	G	G	A
25350	Non-coding	-	C	C	T	C	C	T
25379	Non-coding	-	-	T	-	-	-	-
25381	Non-coding	-	G	G	G	A	G	G
25391	Non-coding	-	G	A	A	A	A	A
25584	CDS	pVIII	G	A	G	A	A	G
25626	CDS	pVIII	C	T	C	T	C	C
25631	CDS	pVIII	T	C	C	C	C	C
25701	CDS	pVIII	A	A	G	A	G	G
25800	CDS	pVIII	T	C	T	C	T	T
25849	CDS	pVIII	A	A	G	A	G	G
25854	CDS	pVIII	T	T	C	T	C	C
26155	Non-coding	-	G	G	T	G	G	T
26195	Non-coding	-	C	C	T	C	C	T
26201	Non-coding	-	G	A	G	A	A	G
26203	Non-coding	-	T	T	C	T	T	C
26269	CDS	E3 19.4K	G	G	A	G	G	A
26283	CDS	E3 19.4K	A	A	G	A	A	G
26294	CDS	E3 19.4K	C	C	T	C	C	T
26306	CDS	E3 19.4K	A	A	G	A	A	G
26317	CDS	E3 19.4K	C	C	A	C	C	A
26528	CDS	E3 19.4K	A	A	A	A	G	A
26649	CDS	E3 19.4K	A	G	A	G	A	A
26830	CDS	E3 31.6K	T	C	C	C	C	C
26953	CDS	E3 31.6K	T	C	T	C	C	T

27350	CDS	E3 31.6K	G	G	A	G	G	A
27435	CDS	E3 31.6K	A	G	A	G	G	A
27450	CDS	E3 31.6K	T	T	T	T	C	T
27451	CDS	E3 31.6K	C	C	C	C	T	C
27533	CDS	E3 31.6K	G	A	G	A	A	G
27751	CDS	E3 10.1K	T	T	T	C	T	T
27829	CDS	E3 10.1K	G	A	G	A	A	G
27876	CDS	E3 14.5K	T	C	T	C	C	T
27887	CDS	E3 14.5K	T	T	T	C	T	T
28134	CDS	E3 14.5K	G	G	G	A	G	G
28225	CDS	E3 14.7K	C	T	C	T	C	C
29081	CDS	short fiber protein	C	C	C	C	A	C
29092	CDS	short fiber protein	A	T	A	T	T	A
29128	CDS	short fiber protein	T	C	C	C	C	C
29150	CDS	short fiber protein	T	G	G	G	G	G
29153	CDS	short fiber protein	A	G	G	G	G	G
29155	CDS	short fiber protein	A	G	G	G	G	G
29179	CDS	short fiber protein	T	C	C	C	C	C
29276	CDS	short fiber protein	A	G	G	G	G	G

29284	CDS	short fiber protein	G	A	A	A	A	A
29591	CDS	short fiber protein	T	C	C	C	C	C
29669	CDS	short fiber protein	A	G	G	G	G	G
29738	CDS	short fiber protein	C	G	G	G	G	G
29743	CDS	short fiber protein	G	A	A	A	A	A
29773	CDS	short fiber protein	C	C	C	C	A	C
29774	CDS	short fiber protein	A	A	A	A	C	A
29866	CDS	short fiber protein	T	C	C	C	C	C
30062	CDS	long fiber protein	A	A	T	A	A	T
30287	CDS	long fiber protein	A	A	T	A	A	T
30318	CDS	long fiber protein	T	T	C	T	T	C
30488	CDS	long fiber protein	T	T	C	T	T	C
30535	CDS	long fiber protein	G	A	A	A	A	A
30608	CDS	long fiber protein	G	A	A	A	A	A
30656	CDS	long fiber protein	T	T	C	T	T	C

30687	CDS	long fiber protein	G	G	T	G	G	T
30728	CDS	long fiber protein	C	C	T	C	C	T
30857	CDS	long fiber protein	T	T	C	T	T	C
30917	CDS	long fiber protein	C	T	C	T	T	C
30932	CDS	long fiber protein	A	G	G	G	G	G
31073	CDS	long fiber protein	C	C	T	C	T	T
31118	CDS	long fiber protein	C	T	C	T	C	C
31121	CDS	long fiber protein	A	A	G	A	G	G
31124	CDS	long fiber protein	A	A	T	A	T	T
31207	CDS	long fiber protein	T	T	T	T	G	T
31262	CDS	long fiber protein	C	G	C	C	C	C
31286	CDS	long fiber protein	T	T	C	T	T	C
31439	CDS	long fiber protein	A	A	A	A	G	A
31441	CDS	long fiber protein	G	G	G	G	T	G
31443	CDS	long fiber protein	T	T	T	T	C	T

31444	CDS	long fiber protein	C	C	C	C	A	C
31451	CDS	long fiber protein	T	T	T	T	C	T
31452	CDS	long fiber protein	C	C	C	C	A	C
31453	CDS	long fiber protein	A	A	A	A	G	A
31454	CDS	long fiber protein	G	G	G	G	A	G
31456	CDS	long fiber protein	A	A	A	A	T	A
31462	CDS	long fiber protein	C	C	C	C	T	C
31463	CDS	long fiber protein	T	T	T	T	C	T
31700	Non-coding	-	C	C	C	C	A	C
31782	Non-coding	-	T	C	C	C	C	C
31893	Non-coding	-	T	T	C	T	T	C
31927	CDS	E4 hypothetical protein 6	C	C	T	C	C	T
31934	CDS	E4 hypothetical protein 6	T	T	C	T	T	C
31969	CDS	E4 hypothetical protein 6	C	C	T	C	C	T
31975	CDS	E4 hypothetical protein 6	C	C	T	C	C	T

31986	CDS	E4 hypothetical protein 6	A	A	G	A	A	G
31991	CDS	E4 hypothetical protein 6	T	T	C	T	T	C
32017	CDS	E4 hypothetical protein 6	C	C	T	C	C	T
32018	CDS	E4 hypothetical protein 6	C	C	T	C	C	T
32042	CDS	E4 hypothetical protein 6	T	T	C	T	T	C
32050	CDS	E4 hypothetical protein 6	C	C	A	C	C	A
32068	CDS	E4 hypothetical protein 6	T	T	G	T	T	G
32073	CDS	E4 hypothetical protein 6	G	G	A	G	G	A
32080	CDS	E4 hypothetical protein 6	T	T	C	T	T	C

32092	CDS	E4 hypothetical protein 6	G	G	A	G	G	A
32098	CDS	E4 hypothetical protein 6	A	A	T	A	A	T
32137	CDS	E4 hypothetical protein 6	A	A	G	A	A	G
32161	CDS	E4 hypothetical protein 6	A	A	G	A	A	G
32185	CDS	E4 hypothetical protein 6	C	T	C	T	T	C
32218	CDS	E4 hypothetical protein 6	G	G	A	G	G	A
32235	CDS	E4 hypothetical protein 6	G	G	A	G	G	A
32245	CDS	E4 hypothetical protein 6	T	T	C	T	T	C
32250	CDS	E4 hypothetical protein 6	A	A	T	A	A	T

32254	CDS	E4 hypothetical protein 6	C	C	T	C	C	T
32263	CDS	E4 hypothetical protein 6	A	G	G	G	G	G
32347	CDS	E4 hypothetical protein 6	T	T	C	T	T	C
32349	CDS	E4 hypothetical protein 6	A	A	G	A	A	G
32377	CDS	E4 hypothetical protein 6	A	A	G	A	A	G
32379	CDS	E4 hypothetical protein 6	G	G	T	G	G	T
32398	CDS	E4 hypothetical protein 6	T	T	C	T	T	C
32469	CDS	E4 hypothetical protein 6	T	T	T	T	C	T
32490	CDS	E4 hypothetical protein 6	T	T	G	T	T	G

32500	CDS	E4 hypothetical protein 6	G	G	A	G	G	A
32525	CDS	E4 hypothetical protein 6	T	T	C	T	T	C
32527	CDS	E4 hypothetical protein 6	G	G	A	G	G	A
32547	CDS	E4 hypothetical protein 6	C	C	T	C	C	T
32553	CDS	E4 hypothetical protein 6	A	A	G	A	A	G
32556	CDS	E4 hypothetical protein 6	C	C	T	C	C	T
32578	CDS	E4 hypothetical protein 6	C	C	T	C	C	T
32592	CDS	E4 hypothetical protein 6	C	C	T	C	C	T
32596	CDS	E4 hypothetical protein 6	A	A	G	A	A	G

32599	CDS	E4 hypothetical protein 6	T	T	A	T	T	A
32615	CDS	E4 hypothetical protein 6	C	C	G	C	C	G
32626	CDS	E4 hypothetical protein 6	A	A	G	A	A	G
32675	CDS	E4 hypothetical protein 6	T	T	C	T	T	C
32686	CDS	E4 hypothetical protein 6	A	A	G	A	A	G
32692	CDS	E4 hypothetical protein 6	A	A	G	A	A	G
32723	CDS	E4 hypothetical protein 6	T	T	C	T	T	C
32730	CDS	E4 hypothetical protein 6	C	C	T	C	C	T
32750	CDS	E4 hypothetical protein 6	C	C	T	C	C	T

32752	CDS	E4 hypothetical protein 6	G	A	G	A	A	G
32783	CDS	E4 hypothetical protein 4	C	C	T	C	C	T
32791	CDS	E4 hypothetical protein 4	T	T	C	T	T	C
32810	CDS	E4 hypothetical protein 4	T	T	C	T	G	C
32856	CDS	E4 hypothetical protein 4	T	C	C	C	C	C
32867	CDS	E4 hypothetical protein 4	T	T	G	T	T	G
32892	CDS	E4 hypothetical protein 4	A	A	G	A	A	G
32897	CDS	E4 hypothetical protein 4	T	T	C	T	T	C
32953	CDS	E4 hypothetical protein 4	T	T	C	T	T	C

32957	CDS	E4 hypothetical protein 4	T	T	C	T	T	C
33017	CDS	E4 hypothetical protein 4	G	G	A	G	G	A
33059	CDS	E4 hypothetical protein 4	T	T	C	T	T	C
33172	CDS	E4 hypothetical protein 3	A	A	G	A	A	G
33216	CDS	E4 hypothetical protein 3	A	A	G	A	A	G
33361	CDS	E4 hypothetical protein 3	A	G	G	G	G	G
33588	CDS	E4 hypothetical protein 2	T	C	C	C	C	C
33636	CDS	E4 hypothetical protein 2	G	G	A	G	G	A
33803	CDS	E4 hypothetical protein 2	A	A	A	C	A	A

33804	CDS	E4 hypothetical protein 2	T	C	C	A	C	C
33842	Non-coding	-	G	C	G	C	G	G
33931	Non-coding	-	G	G	A	G	A	A
33934	Non-coding	-	A	A	G	A	G	G
34007	Non-coding	-	G	-	G	-	-	G
34008	Non-coding	-	C	-	C	-	-	C
34009	Non-coding	-	A	-	A	-	-	A
34010	Non-coding	-	A	-	A	-	-	A
34011	Non-coding	-	A	-	A	-	-	A
34012	Non-coding	-	A	-	A	-	-	A
34013	Non-coding	-	A	-	A	-	-	A
34014	Non-coding	-	A	-	A	-	-	A
34015	Non-coding	-	C	-	C	-	-	C
34016	Non-coding	-	A	-	A	-	-	A
34017	Non-coding	-	C	-	C	-	-	C
34018	Non-coding	-	C	-	C	-	-	C
34019	Non-coding	-	G	-	G	-	-	G
34020	Non-coding	-	C	-	C	-	-	C
34032	Non-coding	-	T	T	A	T	G	G
34064	Non-coding	-	A	G	A	G	A	A
34074	Non-coding	-	A	G	A	G	A	A
34103	Non-coding	-	A	C	A	C	A	A
34112	Non-coding	-	T	A	T	A	T	T
34120	Non-coding	-	A	T	A	T	A	A
34127	Non-coding	-	G	A	G	A	G	G

″-″ means not applicable
